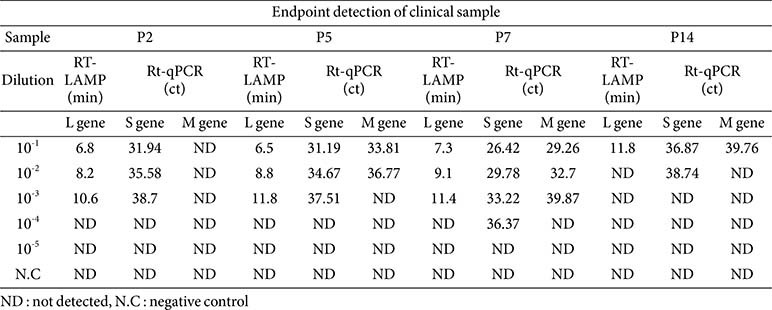# Portable RT-LAMP Platform for Rapid Point-of-Care Detection for SFTSV

**DOI:** 10.4014/jmb.2509.09027

**Published:** 2025-12-09

**Authors:** Gi Chan Lee, Beom Kyu Kim, Sania Batool, Ji-Hyun Park, Seong Cheol Min, Ju Ryeong Lee, Dong Gyu Lee, Se Hee An, Aman Jain, Do Hyung Kim, Hui je Lee, Young Ki Choi, Min-Suk Song, Yun Hee Baek

**Affiliations:** 1 Department of Microbiology, Chungbuk National University College of Medicine and Medical Research Institute, Cheongju, Chungbuk 28644, Republic of Korea; 2 Microuni, Co. Ltd., Cheongju, Chungbuk 28160, Republic of Korea; 3 ELPIS-BIOTECH Company, Deajean 35380, Republic of Korea; 4 Center for Study of Emerging and Re-emerging Viruses, Korea Virus Research Institute, Institute for Basic Science (IBS), Daejeon 34126, Republic of Korea; 5 Chungbuk National University Hospital, Cheongju, Chungbuk 28644, Republic of Korea

**Keywords:** SFTSV, Dabie bandavirus, RT-LAMP, qRT-PCR, diagnosis

## Abstract

Severe Fever with Thrombocytopenia Syndrome virus (SFTSV) is a tick-borne pathogen associated with high mortality, particularly among the elderly, and poses a significant public health threat in East Asia's rural areas. Improved diagnostic approaches are crucial for effective disease management in resource-limited settings. In this study, we developed a portable Reverse Transcription Loop-Mediated Isothermal Amplification (RT-LAMP) platform for rapid and accurate SFTSV detection. Primers targeting the conserved L gene were designed using 158 sequences collected between 2019 and 2023, and a multiplex assay incorporating the β-actin gene as an internal control to verify amplification efficiency in alignment with clinical performance requirements. We assessed the assay’s sensitivity and specificity, ultimately establishing the RT-LAMP platform in a portable, clinical-grade device. The RT-LAMP assay achieved a detection limit of 500 RNA copies per reaction and provided results within 20 min. It demonstrated high specificity without cross-reactivity with other pathogens. Performance comparisons with commercial qRT-PCR kits using clinical specimens confirmed the RT-LAMP platform's reliability. The newly developed RT-LAMP platform is a rapid, accurate, and cost-effective diagnostic tool for SFTSV with strong potential for point-of-care testing (POCT). Its portability and ease of use make it ideal for application in resource-constrained environments, thereby enhancing SFTSV surveillance and improving public health outcomes in affected regions.

## Introduction

Severe Fever with Thrombocytopenia Syndrome Virus (SFTSV), now classified as Dabie bandavirus by the ICTV [1], was first identified in Central China in 2009 [2]. This virus is a member of the Phenuiviridae family and has a tri-segmented, negative-strand RNA genome consisting of large (L), medium (M), and small (S) segments that encode the RNA-dependent RNA Polymerase (RdRp), glycoproteins (Gn and Gc), non-structural protein (NSs), and nucleoprotein (Np). SFTSV is transmitted by ticks and has been reported in six Asian countries: China, Korea, Japan, Vietnam, Myanmar, and Thailand [2-7]. SFTS symptoms typically include fever, fatigue, thrombocytopenia, and leukopenia, with additional symptoms like nausea, headaches, vomiting, diarrhea, muscle soreness, lymph node enlargement, and hemorrhages. Progression can lead to organ failure or diffuse intravascular coagulation, increasing mortality risk, which can be as high as 50% [2].

Point-of-care testing (POCT) is crucial for rapidly diagnosing SFTSV infections, especially in remote areas with limited access to labs. POCT enables healthcare providers to diagnose infections on-site, reducing delays caused by transporting samples. Reverse Transcription Loop-mediated Isothermal Amplification (RT-LAMP) has emerged as a promising tool for POCT due to its efficiency in detecting SFTSV [8, 9]. RT-LAMP amplifies DNA/RNA sequences at a constant temperature, bypassing the complex thermal cycling of quantitative RT-PCR. This simplicity makes RT-LAMP suitable for field use and significantly speeds up the diagnostic process, offering high sensitivity and specificity for detecting SFTSV RNA.

**Fig. 1:**
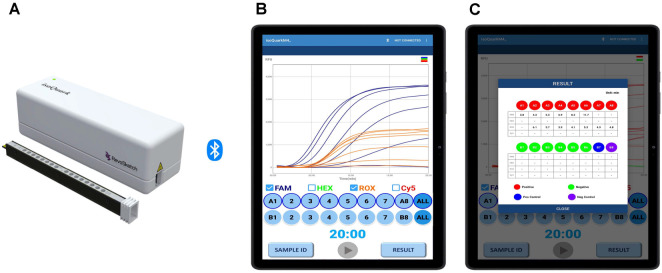
Schematic representation of the use of the portable Revosketch Device (isoQuark M4) in RT-LAMP SFTSV diagnosis. (**A**) isoQuark M4 – Portable Isothermal Device: Illustration of the compact device used for field testing. (**B**) Real-Time Sample Analysis Processing: Depicts the process where data and samples are continuously monitored and analyzed on a connected tablet for a duration of 20 min. (**C**) Result Compilation: Shows the final step where the device compiles the analyzed data and produces conclusive results after the 20-min analysis period.

This study presents a modified RT-LAMP method on a portable isothermal device (Fig. 1). By updating primer sequences and adding a specific probe based on recent genetic analyses, this method enhances test accuracy and reliability, reducing false positives and false negatives. Optimized for affordability and ease of use, it is suitable for primary hospitals or small labs without access to costly equipment. The portable device provides real-time results within 20 min, improving diagnostic efficiency and enabling timely clinical decisions. The method's effectiveness was validated by comparing the sensitivity of RNA extracted from clinical specimens to that of commercial kits.

The growing prevalence of SFTSV highlights the urgent need for accurate diagnostic tools. POCT systems, especially those using RT-LAMP technology in portable devices, offer a promising solution. These systems enhance accessibility, facilitate rapid diagnosis, improve treatment and may ultimately help control the spread of SFTSV.

## Materials and Methods

### Virus Propagation, Titration, and RNA Extraction

Virus isolates of genotype B (43273), C (NCCP 43332), D (NCCP 43265), E (NCCP 43333) were provided by the National Culture Collection for Pathogens (NCCP). The propagation and RNA extraction were conducted in a Biosafety Level 3 (BSL3+) laboratory at the Zoonotic Infectious Diseases Research Center, Chungbuk National University, with approval from the Korea Disease Control and Prevention Agency (KDCA). The viruses were cultured in Vero E6 cells, and titers were quantified using the 50% Tissue Culture Infectious Dose (TCID50) assay, following the Reed and Muench method [10]. RNA was extracted from 250 μl of viral supernatant utilizing the RNeasy Mini Kit (Qiagen, Germany), according to the manufacturer's protocol, and stored at -80°C until required. For RT-LAMP optimization, approximately 1 kb encompassing the RT-LAMP target site of the L gene was cloned using the TOPcloner^TM^ TA kit (Enzynomics, Republic of Korea) and transcribed to generate standard RNA.

### Primer Design for RT-LAMP

A comprehensive analysis of 158 L gene sequences (2019-2023) from the NCBI GenBank repository was conducted using CLC Main Workbench 7 (version 7.6.4) [11-13]. Based on this and a previous study, probes were synthesized for integration with the LAMP primer set (Baek YH *et al*.) [14]. The probes were labeled with 5'-end fluorescent FAM and 3'-end BHQ-1 quencher for complementarity (Table 1). A multiplex RT-LAMP assay was also designed with a primer set targeting the b-actin gene, labeled with ROX and BHQ-2 for detection. The RT-LAMP primer set comprised outer primers (forward outer primer F3 and backward outer primer B3), inner primers (forward inner primer FIP and backward inner primer BIP), and loop primers (forward loop primer LF and backward loop primer LB), designed using Primer Explorer V4 software (http://primerexplorer.jp/elamp4.0.0/index.html). Due to SFTSV genetic variability, modifications were made to ensure primer complementarity, resulting in new primer sequences (Table 1), synthesized by Bionics Co., Republic of Korea.

### Optimization of RT-LAMP Reaction Conditions

The RT-LAMP reaction was performed in a final reaction volume of 20 μl, composed of 10 μl of 2X Enzyme (ELPIS-BIOTECH, Republic of Korea), alongside 8 μl of SFTSV RNA template, 1 μl of SFTS primer mix, 0.5 μl of β-actin (ACTB) gene primer mix, complemented by 0.5 μl of ACTB plasmid DNA (10^5^ copies per well) as an internal control. RT-LAMP experiments were performed utilizing the CFX96 Dx Real-Time PCR Detection System (Bio-Rad, USA) and isoQuark M4 (Revosketch, Republic of Korea) (Fig. 1). The thermal conditions were maintained at 60–65°C for 20 min. The results were analyzed for each gene using the fluorescence channel, with a Ct value (min) below 20 considered positive. However, ACTB detection in the ROX channel might occasionally be reduced due to preferential amplification of the FAM channel (SFTSV) or in cases where amplification is not observed. The isoQuark M4 is a tablet-integrated, real-time isothermal system combining precision heating and four-channel fluorescence (FAM/HEX/ROX/Cy5) with on-device analysis. It maintains 40–70°C at ± 0.3°C; in this study reactions ran at 63°C in 20 μl (8 μl template). The device outputs real-time amplification curves and time-to-positive (min) per user-defined thresholds; results are exportable (CSV/PDF) for QA. The instrument is portable (~0.75 kg), AC-powered, and uses sealed tubes with internal control and NTC in the same run for contamination control and QC.

### Evaluation and Comparison of Sensitivity of RT-LAMP Assays

To assess the sensitivity of the RT-LAMP across different genotypes of SFTSV, a comparative analysis was performed based on the limit of detection (LOD). Synthetic RNA corresponding to the various SFTSV L genes were generated using the Megascript kit (Life Technologies Co. USA) from L gene plasmid clones. This synthetic RNA was then subjected to serial ten-fold dilutions, ranging from 1×10^6^ to 1×10^0^ copies/μl, to ensure precise calibration. These dilutions were utilized in the RT-LAMP to determine and compare the minimum detectable concentrations across various SFTSV genotypes.

### Evaluation and Comparison of Specificity of RT-LAMP Assays

The specificity of the newly developed RT-LAMP diagnostic method was evaluated by testing it against a panel of viruses, including Respiratory Syncytial Virus A and B (RSV-A, RSV-B), Dengue Virus (serotype 2), Zika Virus (MR766), Influenza Virus H1N1, H3N2, CA04, Human enterovirus A71 (EV71), Human coronaviruses OC43, 229E and Heartland virus (HRTV). Viral RNA extractions were performed meticulously using the RNeasy Mini Kit (Qiagen). The RT-LAMP reactions were conducted under carefully optimized conditions to ensure reliability and reproducibility, followed by gene-specific amplification.

### Quantitative Real-Time RT-PCR (qRT-PCR)

The qRT-PCR assays were conducted using the PowerChek SFTSV Real-Time PCR Kit (Kogene Biotech, Republic of Korea) and the CFX96 Dx Real-Time PCR Detection System (Bio-Rad). The reactions were prepared with a total volume of 20 μl, including 5.0 μl of RNA template and 15 μl of PCR mix solution. The qRT-PCR protocol involved an initial reverse transcription step at 50°C for 15 min, followed by a denaturation phase at 95°C for 5 min. The amplification consisted of 45 cycles, with each cycle featuring denaturation at 95°C for 10 sec and annealing at 60°C for 30 sec. A cycle threshold (Ct) value below 40 was indicative of a positive result for detection of the S or M gene.

### Clinical Evaluation

For clinical diagnostic effectiveness, 13 serum and 1 plasma sample from patients suspected with SFTSV infection were kindly obtained from the KBN Human Resource Bank, following approval by the Institutional Review Board (IRB) of Chungbuk National University (CBNU-202402-BR-0289). RNA extraction was performed on all 14 clinical specimens using the QIAamp Viral RNA Mini Kit (Qiagen). RNA was extracted from 140 μl of serum or plasma, eluted in RNase-free water. An aliquot of 5 μl was used in each RT-LAMP and qRT-PCR reaction. The sensitivity and specificity of the RT-LAMP method were then compared to those of the commercially available PowerChek SFTSV Real-time PCR kit (Kogene Biotech). This comparative evaluation aimed to determine the diagnostic performance of the RT-LAMP method relative to established real-time PCR standards, facilitating its potential integration into clinical practice.

## Results

### Optimization and Sensitivity of RT-LAMP Assay for SFTSV Detection

**Fig. 2:**
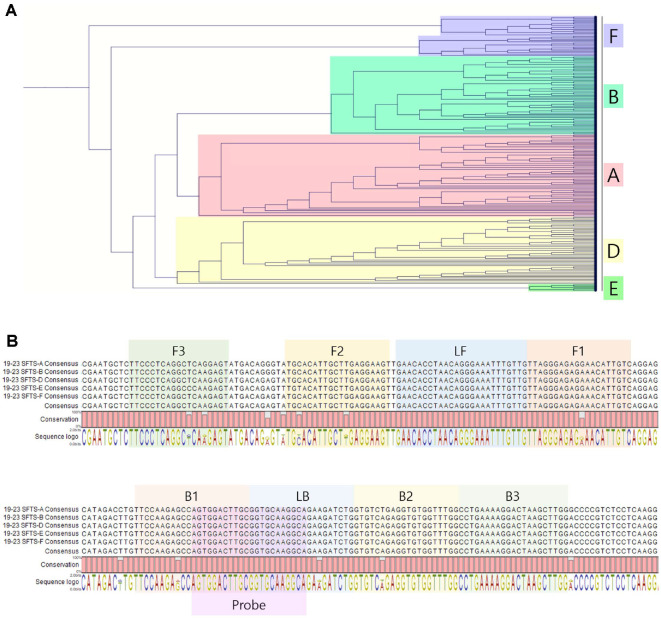
Phylogenetic tree and consensus sequence analysis of SFTSV for RT-LAMP assay primer design. (**A**) Phylogenetic Tree: Displays a phylogenetic tree constructed from the L segment of 158 SFTSV sequences collected between 2019 and 2023, analyzed using CLC Main Workbench 7 (version 7.6.4). This tree highlights the genetic relationships and diversity among the genotypes, except for type C, which was not collected. (**B**) Consensus and Primer Design: Illustrates the consensus sequences for each genotype, excluding type C. Details the design of the RT-LAMP primers used in the assay, including the Forward Inner Primer (FIP), which combines F2 and F1c (F1 complementary sequence), and the Backward Inner Primer (BIP), which combines B2 and B1c (B1 complementary sequence). Forward and backward loop primers (LF and LB, respectively) were specifically designed to enhance the sensitivity of the assay. Refer to Table 1 for detailed information on the SFTSV RT-LAMP primer sequences.

**Fig. 3:**
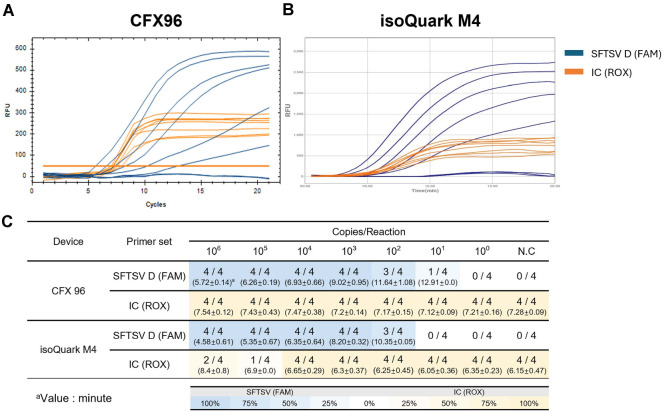
Comparative Sensitivity of Real-time and Portable Isothermal Devices for SFTSV Detection. (**A**) CFX96 Real-Time PCR Detection: This panel shows real-time detection data of SFTSV RNA using the CFX96 device (Bio- Rad). *In vitro* transcribed SFTSV L gene RNA dilutions ranging from 10^6^ to 10^0^ copies per reaction were tested over 20 min. The SFTSV-FAM probe signals are depicted in blue, while the internal control (ACTB-ROX) signals are shown in orange. (**B**) isoQuark M4 Portable Isothermal Detection: Similar to panel A, this panel presents data from the isoQuark M4 device testing the same RNA dilutions. The color coding for SFTSV-FAM and ACTB-ROX remains consistent with blue and orange, respectively. (**C**) Statistical Analysis of Sensitivity Data: Provides average values and standard deviations (SD) from four replicates of the experiments shown in panels (**A**) and (**B**). The results are color-coded to indicate the percentage of positive detection across different RNA copy numbers, enhancing visual comprehension of the comparative sensitivity between the two devices. The y-axis represents relative fluorescence units (RFU). a: Values indicate detection time(minute).

A phylogenetic analysis of 158 SFTSV genomic sequences from GenBank classified distinct genotypes and identified highly conserved regions within the L gene, essential for designing RT-LAMP primers (Fig. 2 and Table 1). For RT-LAMP optimization used *in vitro* transcribed genotype D RNA, with amplification tests performed using 10^3^ and 10^4^ copies of synthetic SFTSV RNA and β-actin (ACTB) as an internal control. Reactions were conducted at 61–65°C and varying probe concentrations on the CFX96 system, identifying optimal conditions at 63°C with a probe ratio of 1:0.25. These parameters were used in subsequent evaluations (Fig. S1). Under these conditions, the RT-LAMP consistently detected the presence of as few as 10^2^ RNA copies in over 75%of attempts using both the CFX96 and isoQuark M4 device (Fig. 3). Extensive validation involved 20 repetitions at 1000, 500, 250, and 100 RNA copies per reaction showed 95% and 100% positivity for 500 RNA copies with the CFX96 and isoQuark M4, respectively (Table 2). For 250 RNA copies, positivity was 85% and 75%, respectively. The minimum detectable limit, demonstrating a 95% detection efficiency, was established at 500 RNA copies per reaction for both systems.

### Detection across Various SFTSV Genotypes

Given the diversity of SFTSV genotypes, the cross-detectability of the optimized RT-LAMP assay using the isoQuark M4 device was evaluated. Viral RNA from SFTSV genotypes B, C, D, and E, adjusted to a titer of 3.5 log TCID_50_/ml, was extracted following propagation and subjected to 10-fold serial dilutions down to 10^-7^. The RT-LAMP amplified RNA within 20 min, achieving 100% detection of genotype D at 10^-7^ dilution (Table 3). For genotypes C and E, detection reached 50% at 10^-7^, while genotype B was detected in 25% of tests at 10^-6^. Additional testing was performed using synthetic RNAs, transcribed from plasmid clones of the L gene corresponding to SFTSV genotypes B, C, D, and E, subjected to similar 10-fold serial dilutions. These tests mirrored the sensitivity observed with real viral RNA, as documented for the respective genotypes (Table S1). This consistent detection across both real and synthetic RNA samples demonstrates the high and broad cross-reactivity of the RT-LAMP among different SFTSV genotypes, highlighting its effectiveness for diverse diagnostic applications.

### Specificity of the RT-LAMP Assay for SFTSV Detection among Other Human Infectious Viruses

To validate the RT-LAMP assay’s specificity, RNA was extracted from 10 different human infectious viruses that exhibit clinical symptoms similar to SFTSV, such as high fever. The SFTSV-specific RT-LAMP test was subsequently performed on these extracted samples While ACTB gene amplification (internal control) was confirmed in all samples, a positive result appeared only in the SFTSV-positive control (Table 4). Importantly, no amplification of the SFTSV gene occurred in any of the samples from the other ten viruses, confirming that the developed RT-LAMP assay specifically amplifies the SFTSV gene without cross-reactivity to other infectious viruses. This result underscores the assay's high specificity, making it a reliable tool for diagnosing SFTSV in the presence of other febrile illnesses.

### Evaluation of RT-LAMP Assay Using Clinical Samples in Comparison with a Commercial SFTSV qRT-PCR Kit

To assess the RT-LAMP assay’s effectiveness in detecting SFTSV infection, RNA was extracted from 14 clinical samples (serum and plasma) from suspected SFTSV patients. The RT-LAMP was compared with a commercially available qRT-PCR kit, which targets the S and M gene, using undiluted RNA extracts. Both methods detected positive reactions in 4 out of the 14 samples, showing comparable efficacy (Table 5). Further evaluation was conducted by performing a 10-fold serial dilution on the four samples that tested positive (P2, P5, P7, and P14) to compare the sensitivity of the RT-LAMP and qRT-PCR methods. Both methods successfully detected samples P2 and P5 up to a dilution level of 10^-3^, although the qRT-PCR detection of the M gene failed in sample P2 and detected only up to 10^-2^ in sample P5. For sample P7, the RT-LAMP assay detected the virus up to 10^-3^, whereas the qRT-PCR detected the S gene up to 10^-4^ and the M gene up to 10^-3^. Sample P14 was detected by RT-LAMP up to a dilution of 10^-1^, whereas the qRT-PCR detected the S gene up to 10^-2^ and the M gene up to 10^-1^ (Table 6). These findings suggest that the RT-LAMP assay's sensitivity is comparable to qRT-PCR, making RT-LAMP a viable alternative for rapid on-site diagnosis of SFTSV.

## Discussion

SFTSV is an emerging tick-borne pathogen with a high mortality rate, making early and accurate diagnosis essential for effective disease management. We introduced and validated a tablet-based RT-LAMP platform for rapid SFTSV detection, offering a portable and user-friendly diagnostic solution for resource-limited settings.

The isoQuark M4 device, with KMDIS (Korea Medical Devices Information Search system) certification and CE (Conformité Européenne) approval, ensures reliable, standardized performance for clinical use [15, 16]. The RT-LAMP assay demonstrated high sensitivity and specificity, achieving a detection limit of 500 RNA copies per reaction with both CFX96 and isoQuark M4 devices. It detects various SFTSV genotypes within 20 min, and for samples with over 1,000 RNA copies, detection occurs in 10 minutes in a real-time manner, facilitating timely clinical interventions. Our results are consistent with previous RT-LAMP studies on SFTSV, which reported detection limits of 500 RNA copies [17] and 100 plasmid copies within 20 min [9]. Specificity was confirmed by testing against RNA from ten other infectious agents, with no cross-reactivity, ensuring accurate diagnoses. Negative controls showed no false positive, confirming the assay's specificity. We selected the L segment (RdRp) as the RT-LAMP target because it shows the highest intra-genotype conservation in SFTSV, whereas the M (glycoprotein) and S (NP/NSs) segments are more variable and prone to immune/segmental diversity. Given the phylogenetic relatedness to Heartland virus (HRTV), we additionally assessed inter-species similarity. Whole-L alignments demonstrated marked divergence from non-SFTSV bandaviruses (*e.g.*, HRTV; ~68% nucleotide identity across L), and-critically-multiple mismatches within our primer/probe windows (including 3'-terminal sites) in non-target taxa, supporting assay exclusivity under isothermal conditions (Fig. S2).

In comparison with existing SFTSV RT-LAMP approaches, dye-based readouts [17] are simple but prone to non-specific signal and are less suitable for multiplexing/internal controls, whereas probe-based formats, Qprobe relies on guanine-dependent quenching with an inverted (signal-decrease) readout that limits multiplexing [8], while DARQ enables signal-increase detection and multiplexing but often requires multiple custom oligonucleotides and careful probe-ratio optimization [9]. Our assay employs a sequence-specific dual-labeled quenched probe for SFTSV with a ROX-labeled ACTB internal control in a true two-channel multiplex on a portable real-time device, providing higher signal-to-noise, straightforward design, and on-device amplification curves optimized for POCT. Relative to our earlier 2018 JMB report [14], the present platform introduces a true two-channel multiplex (SFTSV-FAM plus ACTB-ROX) with a built-in internal control—absent in the earlier version—thereby enabling verification of negative calls and run-level quality control. It also integrates real-time fluorescence curve monitoring on a tablet-based portable device, improving adjudication of equivocal results and field deployability for POCT.

To contextualize analytical performance clinically, our LOD of 500 copies/reaction (using 8 μl input in a 20 μl reaction) corresponds to ~6.25 × 10^4^ copies/ml. Reported SFTSV loads typically peak in early acute/severe disease (often ≥10^5^–10^6^ copies/ml), whereas mild/asymptomatic or convalescent phases frequently fall in the 10^3^–10^4^ copies/ml range (*e.g.*, recent cohort data) [18]. Thus, the platform is well-suited for rapid triage of acute/high-titer cases at the point of care, while very low-titer presentations may approach or fall below the LOD.

In settings of high clinical suspicion with a negative result, we recommend confirmation by qRT-PCR. Sensitivity for low-titer samples can be further supported by increasing template input or concentrating extracts (larger sample volume with smaller elution), and by replicate testing. Clinical evaluations underscored the assay's practical applicability, showing consistent detection of SFTSV in patient samples, comparable to results from a commercial qRT-PCR kit. In other studies, RNA extracted from clinical specimens and compared with the PowerChek SFTSV real-time PCR kit showed similar detection levels for the monoplex assay, but the multiplex assay had a 4-fold reduction in sensitivity [8].

In our study, the devicés portability and simple tablet interface make it ideal for remote and smaller healthcare settings. While other studies like colorimetric, on-chip, and cellphone-based methods enhance convenience, they struggle with monitoring quantitative changes in real-time. In contrast, our device not only enables rapid detection but also supports real-time amplification curve monitoring, ensuring improved accuracy and reliability [19-21]. Especially, colorimetric LAMP methods can suffer from subjective interpretation due to pH fluctuations causing non-specific color changes, and their sensitivity is limited. whereas, probe-based methods offer higher specificity, making them more reliable for clinical applications [14]. This interface facilitates real-time monitoring and result compilation, simplifying testing and reducing the need for specialized training. Furthermore, incorporating updated primer and a fluorescent probe enhances accuracy, reducing false positives and negatives. Given SFTSV’s high fatality rate, rapid and reliable diagnostic tools are paramount. The RT-LAMP platform supports the rapid identification and isolation of infected individuals, offering significant advantages during outbreaks. RNA extraction is still required, but simpler methods, such as RNAGEM (MicroGEM, UK), are used in field diagnostics. Another study compared direct RT-LAMP with extracted RNA-based RT-LAMP and qRT-PCR, showed 91.7% and 88.3% agreement rates, respectively. Adapting this approach to our kit could enable quicker virus screening for POCT, with serum separation, heat treatment, and RT-LAMP facilitating on-site diagnostics [22].

In conclusion, the tablet-based RT-LAMP platform advances POCT for SFTSV. This platform can improve patient outcomes and public health responses through its rapid, sensitive, and specific detection capabilities. Future research should refine the RT-LAMP assay's robustness against genetic variations in SFTSV and other emerging pathogens. Extensive field studies are needed to validate their effectiveness in diverse environments and exploring its multiplexing capabilities to detect multiple pathogens simultaneously would further enhance its utility.

## Supplemental Materials

Supplementary data for this paper are available on-line only at http://jmb.or.kr.



## Figures and Tables

**Table 1:** Multiplex RT-LAMP primer/probe sequences for the L gene of SFTSV and internal control (β-actin).

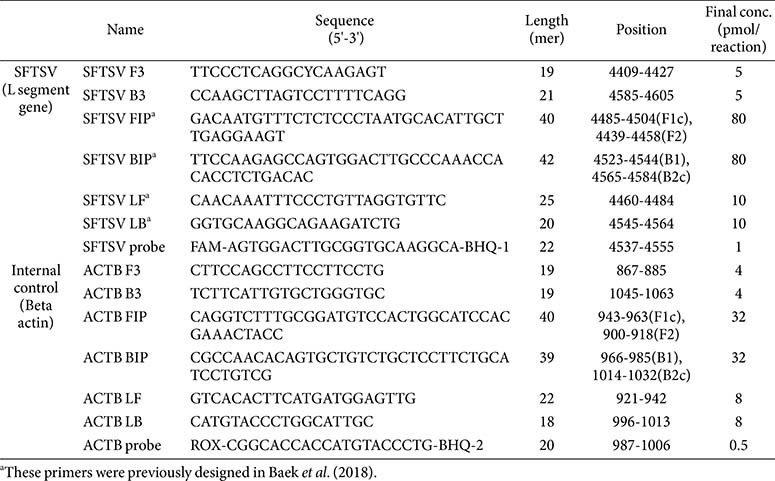

**Table 2:** Comparative analysis of detection limits between the real-time PCR device (CFX96) and portable isothermal device (isoQuark M4).

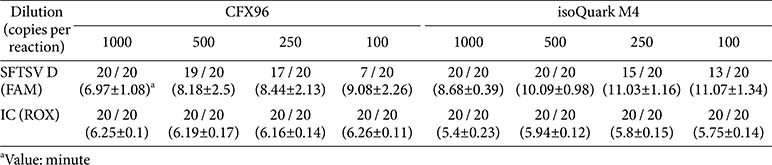

**Table 3:** Evaluation of virus genotype Detection by isoQuark M4.

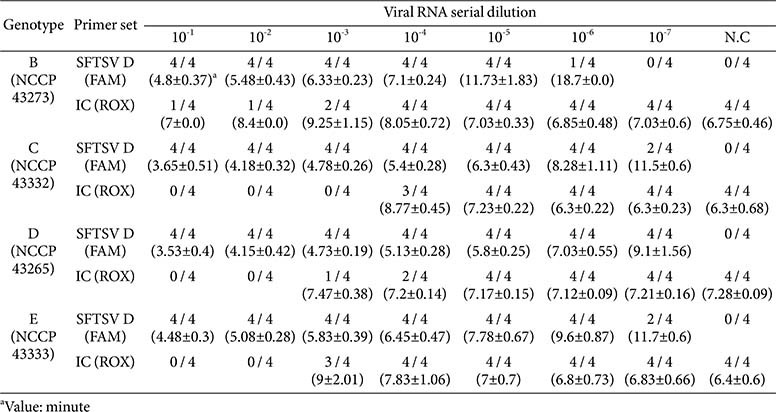

**Table 4:** Cross-reactivity of the multiplex SFTSV RT-LAMP as compared to other infectious viruses.

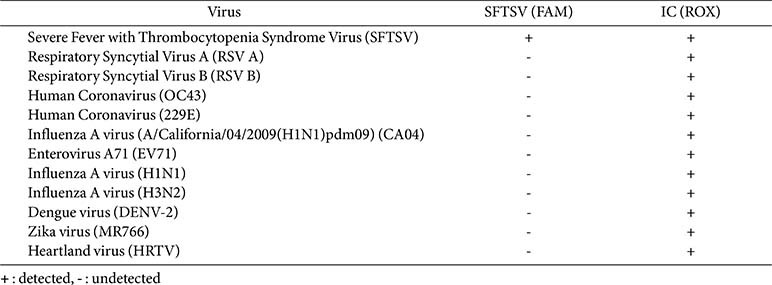

**Table 5:** Evaluation of RT-LAMP assay and RT-qPCR kit using clinical samples.

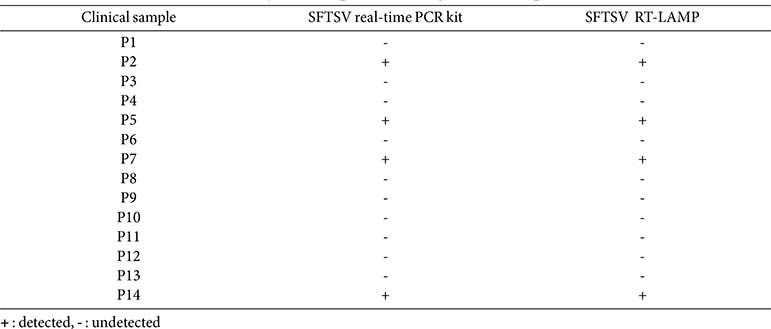

**Table 6:** Comparative analysis of clinical samples using SFTSV RT-LAMP and PowerChek SFTSV real-time PCR kit.