# Supplementary material for: Portable RT-LAMP Platform for Rapid Point-of-Care Detection for SFTSV
Source: J Microbiol Biotechnol. 2025 Dec 9;35:e2509027. doi: 10.4014/jmb.2509.09027 (PMC12706147; doi:10.4014/jmb.2509.09027)
Supplement: Supplementary file 1 [file jmb-35-e2509027-supple.pdf]

J. Microbiol. Biotechnol. 2025. 35: e2509027

https://doi.org/10.4014/jmb.2509.09027

Supplementary Figures and Table

Portable RT-LAMP Platform for Rapid Point-of-Care Detection for SFTSV

Gi Chan Lee[1,2], Beom Kyu Kim[1,2], Sania Batool[1], Ji-Hyun Park[1], Seong Cheol Min[1], Ju Ryeong Lee[1], Dong Gyu Lee[1], Se Hee An[1], Aman Jain[1], Do-Hyung Kim[3], Hui je Lee[3], Young Ki Choi[4], Min-Suk Song[1,2], Yun Hee Baek[1]

[1]Department of Microbiology, Chungbuk National University College of Medicine and Medical Research Institute, Cheongju, Chungbuk, 28644, Republic of Korea.

[2]Microuni, Co. Ltd., Cheongju, Chungbuk, Republic of Korea.

[3]ELPIS-BIOTECH Company, Deajean, Republic of Korea.

[4]Center for Study of Emerging and Re-emerging Viruses, Korea Virus Research Institute, Institute for Basic Science (IBS), Daejeon, Republic of Korea

[†]Contributed equally.

*Corresponding Authors:

Min-Suk Song (E-mail: songminsuk@chungbuk.ac.kr)

Yun Hee Baek (E-mail: microuni@chungbuk.ac.kr)

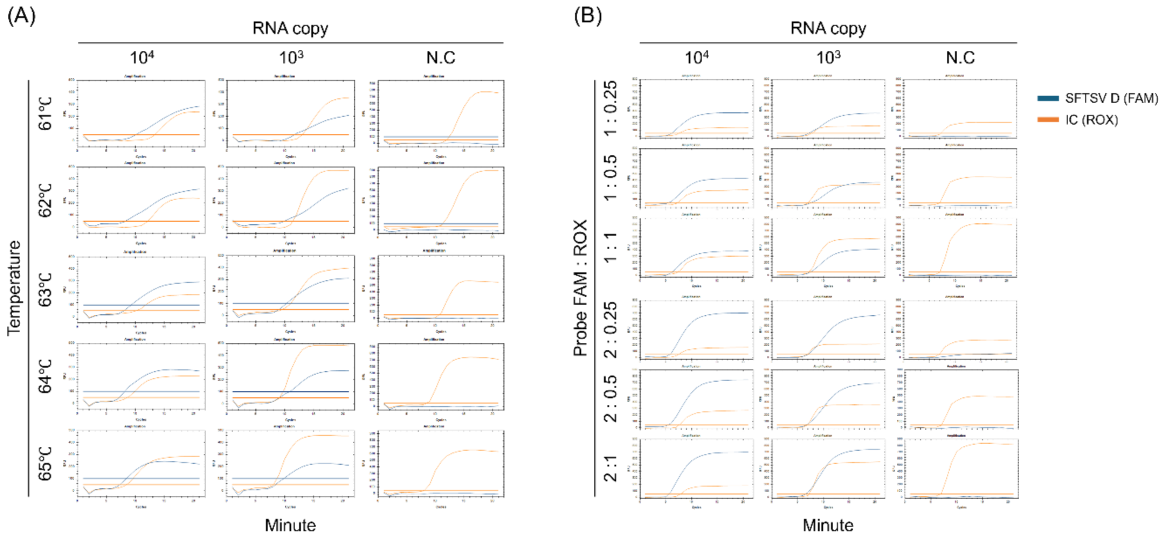

Fig. S1. Optimization of the Multiplex RT-LAMP Primer Set for SFTSV and Internal Control (IC). (A) Temperature Gradient Test: A temperature gradient test was performed, ranging from 61°C to 65°C, to optimize conditions for the multiplex RT-LAMP assay targeting both SFTSV and the internal control (IC). **(B)** Probe Concentration Ratio Optimization: This panel displays the results from testing different concentration ratios of SFTSV and IC probes. Ratios tested include 1:0.25, 1:0.5, 1:1, 2:0.25, 2:0.5, and 2:1 (FAM for SFTSV and ROX for IC), conducted at an optimized temperature of 63°C. Probe signals for SFTSV-FAM are indicated, alongside IC-ROX, to evaluate the efficiency of different probe combinations in the assay. N.C, non-template control.

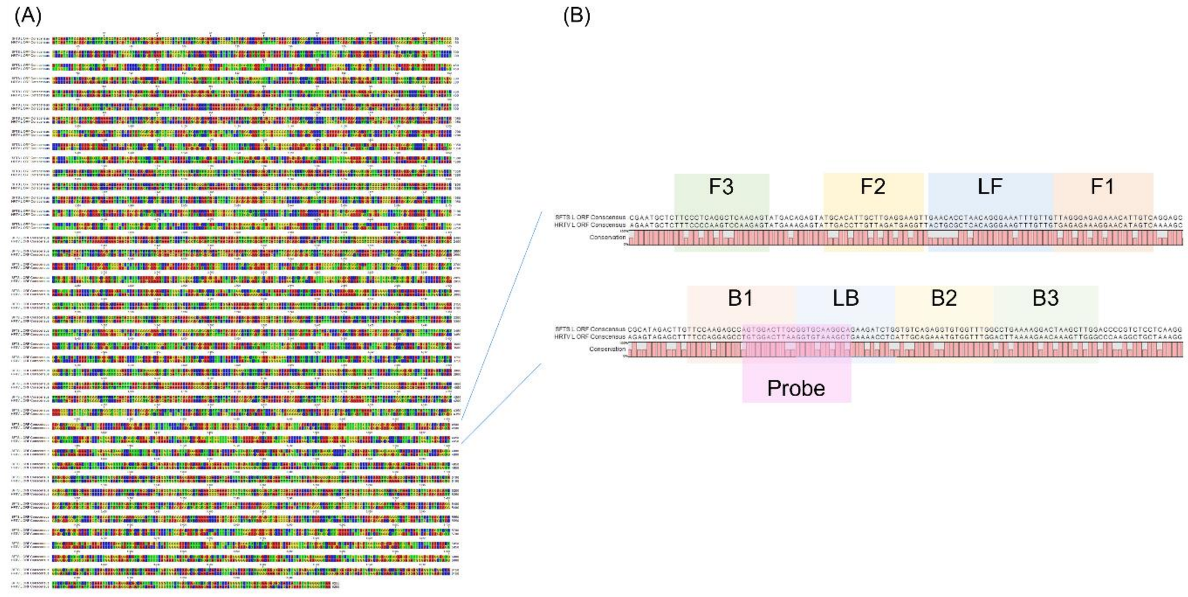

Fig. S2. Nucleotide comparison of the L gene between SFTSV and HRTV. (A) Full-length alignment of the L gene between SFTSV and HRTV shows an overall 68.3% nucleotide identity, with sequence mismatches distributed throughout the region. **(B)** Magnified view of the RT-LAMP target region. Multiple nucleotide mismatches, particularly within primer-binding sites, support the specificity of the assay. Refer to Table 1 for detailed information on the SFTSV RT-LAMP primer sequences.

Table S1. Sensitivity Analysis of SFTSV RT-LAMP Primers Using In Vitro Transcribed RNA from Various L Gene Clones Detected by the isoQuark M4 Device.

Genotype	Primer set	RNA transcript Copy Number							
		8 x 10^6	8 x 10^5	8 x 10^4	8 x 10^3	8 x 10^2	8 x 10^1	8 x 10^0	N.C
B (NCCP 43273)	SFTSV	+ / +	+ / +	+ / +	+ / +	+ / -	- / -	- / -	- / -
	I.C	+ / -	+ / +	+ / +	+ / +	+ / +	+ / +	+ / +	+ / +
C (NCCP 43332)	SFTSV	+ / +	+ / +	+ / +	+ / +	+ / +	+ / +	- / -	- / -
	I.C	+ / +	+ / +	+ / +	+ / +	+ / +	+ / +	+ / +	+ / +
D (NCCP 43265)	SFTSV	+ / +	+ / +	+ / +	+ / +	+ / +	- / -	- / -	- / -
	I.C	- / -	+ / +	+ / +	+ / +	+ / +	+ / +	+ / +	+ / +
E (NCCP 43333)	SFTSV	+ / +	+ / +	+ / +	+ / +	+ / +	- / -	- / -	- / -
	I.C	+ / +	+ / +	+ / +	+ / +	+ / +	+ / +	+ / +	+ / +

+ : detected, - : undetected
